# Psychosocial Determinants of Health Behaviour Change in an E-Counseling Intervention for Hypertension

**DOI:** 10.1155/2012/191789

**Published:** 2011-12-20

**Authors:** Samir Durrani, Jane Irvine, Robert P. Nolan

**Affiliations:** ^1^Department of Psychology, York University, 4700 Keele Street, Toronto, ON, Canada M3J 1P3; ^2^Behavioural Cardiology Research Unit, University Health Network, 585 University Avenue, Toronto, ON, Canada M5G 2N2; ^3^Department of Psychiatry, University of Toronto, 1 Kings College Circle, Toronto, ON, Canada M5S 1A8

## Abstract

We evaluated the influence of psychological stress and depression on motivation to adhere to recommended guidelines for exercise and diet. This study was conducted within a larger e-counseling trial. Subjects diagnosed with hypertension (*n* = 387, age = 44–74 years, 59% female) completed assessments at baseline and within 2 weeks after a 4-month intervention period. Outcomes included mean level of readiness to change diet and exercise and symptoms of depression and stress. Per protocol analysis defined e-counseling support as follows: ≥8 e-mails = therapeutic dose, 1–7 e-mails = subtherapeutic dose, and 0 e-mails = Controls. Baseline adjusted symptoms of depression and stress were inversely correlated with improvement in exercise (partial *R* = −.14, *P* = .01, and partial *R* = −.17, *P* = .01, resp.) but not diet or e-counseling. Subjects who received a therapeutic dose of e-counseling demonstrated greater readiness for diet adherence versus Controls (*P* = .02). Similarly, subjects receiving a therapeutic level of e-counseling demonstrated significantly greater readiness for exercise adherence versus Controls (*P* = .04). In sum, e-counseling is associated with improved motivation to adhere to exercise and diet among patients with hypertension, independent of symptoms of psychological stress and depression.

## 1. Introduction

Health behaviour change for diet and exercise in combination with drug therapy is critical for the treatment of hypertension [1]. Meta-analysis indicates that counseling initiatives to increase patient motivation to change health behaviour can significantly improve outcomes across a wide array of behaviours, including exercise and diet [2, 3]. However, it is not established whether novel programs of motivational counseling, which are delivered via the internet, are sufficient to improve patient readiness to adhere to exercise and diet, among persons diagnosed with hypertension. It is also unknown whether the potential efficacy of this e-counseling initiative is compromised significantly by symptoms of psychological stress and depression, which are well known to inhibit motivation to change health behaviours [4, 5].

The I-START trial (Internet-based Strategic Transdisciplinary Approach to Risk Reduction and Treatment) was an e-counseling intervention that used goal setting features from motivational interviewing [6] to improve adherence to health behaviour change in patients diagnosed with hypertension. The purpose of the present substudy was to evaluate whether e-counseling significantly increased motivation to change exercise and diet behaviour in patients enrolled in I-START, as defined by the Transtheoretical model of stages of readiness for change [7, 8]. In addition, this study investigated whether behaviour change following e-counseling in I-START was significantly associated with presenting symptoms of psychological stress and depression.

## 2. Methods

### 2.1. Subjects

Subjects were recruited by self-referral through an e-based program of the Heart and Stroke Foundation of Canada (HSF): My Blood Pressure Action Plan (BPAP); http://www.heartandstroke.ca/hs_Risk.asp?media=bp_hsfhomepage.

### 2.2. Inclusion/Exclusion Criteria

Enrollment included 387 subjects between the ages of 45–74 years in this study. All subjects were diagnosed with stage I or II hypertension (140–159/90–99 mmHg or 160–180/100–110 mmHg, resp.) as confirmed by their family physician. Subjects receiving antihypertensive pharmacotherapy were required to have had an unchanged treatment regimen for a minimum of 4 months. Participation of these subjects was also contingent on confirmation by their physician that there was no current plan to alter their medical treatment. Subjects were not living in an institution and they were competent in English or French. All subjects provided consent to consult with their family physician to confirm medications and diagnosis.

Subjects were excluded from the study if they self-reported having a current diagnosis of cardiovascular disease or major psychopathology (e.g., bipolar mood disorder), or a history of alcohol or substance abuse in the last year, or if they had an anxiety disorder due to panic, phobias, obsession-compulsion, or traumatic stress.

### 2.3. Materials

Readiness to change health behaviour was assessed using a health promotion lifestyle profile (HPLP) [9]. This profile provided a global index of adherence according to Health Canada Guidelines for exercise, diet, and smoke-free living as defined by the self-reported stage of readiness to change the following behaviours: daily dietary intake of vegetables and fruits, dietary restriction of fat and sodium, daily physical activity and weekly planned exercise, as well as smoke-free living. The HPLP measured stage of readiness to change on a 4-point continuous scale (Table 1) in which 1 corresponded to the Precontemplation stage, 2 corresponded to the Contemplation stage, 3 corresponded to the Preparation stage, and 4 corresponded to the Action/Maintenance stage. The HPLP is inversely associated with body weight, Body Mass Index, waist circumference, and changes in weight over time [9].

Symptoms of depression were assessed using the Beck Depression Inventory-II (BDI-II), which is a 21-item self-administered inventory designed to measure cognitive-affective and somatic-behavioural symptoms of depression present in the last two weeks [10].

 Psychological stress was measured using the Perceived Stress Scale (PSS), which is a self-administered scale that assesses the degree to which situations in one's life during the last month are appraised as stressful [11].

### 2.4. Procedure

Participants were first given a baseline assessment in which anthropometric data, stage of readiness to change health behaviours, depression, and psychological stress were evaluated. Following assessment, participants were randomized into two conditions: the 4-month e-Counseling condition, using the HSF web-based program entitled “Blood Pressure Action Plan” (BPAP) [12] and a Waitlist Control condition. Subjects in the e-Counseling group identified their priority for behaviour change from a list of recommended lifestyle changes for blood pressure control. They self-rated their motivation to initiate/maintain change for this behaviour, and the e-counseling system provided them with programmed feedback on “change goals” that were based on principles from motivational interviewing [6] and were tailored to each individual's level of readiness for health behaviour change. They were sent weekly e-mails during the first month, biweekly e-mails during the second month, and monthly e-mails during months 3 and 4. Controls received an e-health newsletter from the HSF that contained general information and advice for cardiovascular health and healthy living. The Post-Intervention assessment included the full assessment protocol that was administered at Baseline, and it was scheduled within 2 weeks following completion of the 4-month intervention.

All subjects provided informed consent prior to participating in the study, and the study was approved by the Research Ethics Board of all participating institutions.

### 2.5. Statistical Analysis

For the purpose of this substudy of our clinical trial, the results are presented according to a per protocol analysis that includes 3 groups: e-counseling subjects who received ≥8 e-mails (therapeutic dose); e-counseling subjects who received 1 to 7 e-mails (subtherapeutic dose); and subjects who received 0 e-mails (control condition).

Descriptive analyses and analysis of variance or chi-square analyses were used to describe sample characteristics between the three groups. Analysis of covariance was used to evaluate the effectiveness of e-counseling in improving levels of readiness to change exercise and diet behaviour, as well as levels of depression, and stress, adjusted for baseline readiness to change exercise and diet, baseline depression and stress, sex, age, and antidepressant-anxiolytic medications. Paired *t*-tests were used to examine the main effect of change in psychological distress over time. Bonferroni post hoc tests (adjusted for two post hoc comparisons) were used to compare both e-Counseling groups with the Control group. Zero-order and partial correlations were used to examine the association between psychological variables and readiness for health behaviour change. Partial correlations were adjusted for baseline stress and depression, as well as baseline exercise and diet.

## 3. Results

Initially, 10, 658 individuals who resided within the recruitment areas for the present study completed the self-assessment program on the website for HSF. From this sample, 782 individuals completed the telephone screening for this investigation. The present study sample enrolled 387 subjects who met the eligibility criteria and who provided written consent to participate in the study.

### 3.1. Baseline Characteristics of Sample

Table 2 presents the background characteristics of the sample measured at baseline, grouped according to our per protocol analysis of therapeutic e-counseling (≥8 e-mails), subtherapeutic e-counseling (1–7 e-mails), and the control condition (0 e-mails). There were no significant differences between groups in age, income tertile, education, Framingham ten-year absolute risk score, CVD risk factors, stress, depression, readiness to change exercise, and readiness to change diet. There was a significant group difference in sex, in which there was a greater percentage of female subjects in the therapeutic e-counseling groups (*χ* ^2^ = 10.7, *P* = 0.005).

### 3.2. Health Behaviour Change and Psychological Distress

Table 3 displays the zero-order correlations between baseline levels of psychological distress and readiness for health behaviour change. Partial correlations are also presented for baseline-adjusted scores for stress and depression and self-reported exercise and diet at the Post-Intervention assessment. At baseline, stress and depression were inversely associated with readiness to change exercise (*P* = 0.001 and *P* = 0.002, resp.) and readiness to change diet (*P* = 0.04 and *P* = 0.05, resp.). There was also a significant inverse association between baseline adjusted stress and depression with exercise at Post-Intervention (*P* = 0.01 and *P* = 0.01, resp.), but not for diet.

### 3.3. Psychological Distress, E-mail Support, and Health Behaviour Change

Table 4 displays the mean values for readiness to change exercise and diet behaviour at the Post-Intervention assessment. There was a significant difference among the group means for both readiness to change exercise and diet behaviour (*F* = 5.55, *P* = 0.02, partial *η* ^2^ = .02 and *F* = 5.07, *P* = 0.03, partial *η* ^2^ = .02, resp.). Post hoc comparisons demonstrated that the mean for readiness to change exercise and the mean for readiness to change diet in the therapeutic e-counseling group was significantly greater than the mean for Controls.

There were no significant group differences in mean levels of stress (*P* = 0.96) or depression (*P* = 0.35) at Post-Intervention, after adjusting for baseline stress and depression, as well as antidepressant-anxiolytic medications. Nevertheless, we observed a main effect for change in means for stress and depression across baseline and Post-Intervention assessments, independent of our intervention: depression, 7.9 ± 0.04 and 6.1 ± 0.04, *P* < .001; stress, 14.8 ± 0.03 and 13.6 ± 0.03, *P* < .001.

## 4. Discussion

The results of this study demonstrate that e-counseling is significantly associated with therapeutic improvements in lifestyle change. A reduction in symptoms of depression and stress was significantly associated with an increase in readiness to change exercise. Interestingly, readiness to change diet was not associated with change in levels of depression and stress which suggested that this relationship may be mediated by situational factors outside the scope of this analysis. To our knowledge, this is the first study to show that e-counseling with features of motivational interviewing [6] is significantly associated with improved readiness to change exercise and diet behaviour among patients with hypertension, independent of symptoms of psychological distress.

### 4.1. Psychological Distress and Health Behaviour Change

Baseline stress and depression were inversely associated with baseline levels of readiness to change exercise and diet. This is consistent with previous reports that psychological distress is a barrier to health behaviour change which in turn can impede blood pressure control [1]. It is noteworthy that our findings partially corroborate this theory. Change in stress and depression over the 4-month intervention period was inversely associated with exercise at Post-Intervention. On the other hand, we failed to observe an association between Post-Intervention assessment of diet and corresponding levels of psychological stress and depression. We additionally failed to observe a direct effect of e-counseling on these symptoms of psychological distress. Nevertheless, we did observe a main effect of change in these symptoms across baseline and Post-Intervention assessments, independent of the intervention. Given that stress reduction is commonly recommended for patients with hypertension who also present with elevated psychological distress, it is a priority for future trials to establish whether the combination of e-counseling for lifestyle and stress reduction can evoke a direct or indirect therapeutic effect on blood pressure control.

It is important to note that the current findings do not clarify the causal relationship between psychological distress and motivation to change exercise and diet. It is possible that as subjects were enrolled in this study their symptoms of stress and depression decreased, which in turn facilitated an increase in motivation to adhere to health behaviours. Alternatively, increased adherence to health behaviours may have facilitated a reduction in symptoms of stress and depression. Indeed, the relationship between psychological distress and health behaviour is likely bidirectional in nature [13].

This study did not find a significant relationship between psychological distress and readiness to change diet at the Post-Intervention assessment, suggesting a more complex relationship between these variables. As others have suggested [14], dietary behaviour is determined by biological, anthropological, economic, psychological, and socio-cultural factors. It may be advisable for future e-counseling initiatives for hypertension to monitor social determinants of adherence to diet since these may be important therapeutic targets for intervention.

### 4.2. Limitations

The findings of this study may be limited to subjects who have relatively low levels of psychological distress and increased motivation to pursue self-help information for improving heart health. Subjects enrolled in the present study had initially logged onto the BPAP program of the Heart and Stroke Foundation website. In addition, measures of readiness to change are self-report and may have been influenced by socially desirable responding. Nevertheless, we have presented preliminary evidence supporting the criterion validity of the self-reported readiness to change exercise and diet. We reported that these measures of readiness were inversely associated with body weight, Body Mass Index, waist circumference, and changes in weight over time [9].

## 5. Conclusion

A novel finding of the present study is that e-counseling is associated with improved motivation to adhere to exercise and diet among patients with hypertension, independent of symptoms of psychological stress and depression. Given that we observed an inverse relationship between psychological distress and self-reported exercise following the intervention, a priority for future research is to determine whether e-counseling for lifestyle and stress reduction offer a therapeutic advantage for blood pressure control.

## Figures and Tables

**Table 1:** Stage of readiness to change health behaviour.

Scale Number	Stage of Change	Definition
(1)	Precontemplation	Having little or no intention of changing behaviour in the next 6 months.
(2)	Contemplation	Beginning to acknowledge that they have a problem, and thinking seriously about solving it; intention to change within 6 months.
(3)	Preparation	Making plans about how they will change their behaviour within the next month.
(4)	Action/Maintenance	Making overt changes in their behaviour and surroundings in the last 6 months and/or maintaining behaviour change, often with relapses; can last from 6 months to about 5 years.

Note. Definition of Stage of Change is based on Prochaska's Stages of Change Model [8].

**Table 2:** Baseline characteristics of subjects.

Characteristics	M ± SE or *N* (%)			
	0 e-mails (*n* = 227)	1–7 e-mails (*n* = 63)	≥8 e-mails (*n* = 97)	*P* value
Age (years)	56.7 ± 0.5	57.0 ± 0.9	55.6 ± 0.7	0.40
Sex: Female (%)	120 (52.9)	39 (61.9)	70 (72.2)	0.005

Household income:				
<$60k	80 (35.2)	16 (25.4)	32 (33.0)	0.63
$60 k–$99,999	81 (35.7)	24 (38.1)	37 (38.1)	
≥$100 k	66 (29.2)	23 (36.5)	28 (28.9)	

Body mass index (kg/m^2^)				
<25	41 (18.1)	11 (17.5)	18 (18.6)	0.85
25–29.9	88 (38.8)	29 (46.0)	41 (42.3)	
≥30	98 (43.2)	23 (36.5)	38 (39.2)	

Waist circumference				
*F* > 88 cm, *M* > 102 cm	143 (63.0)	36 (57.1)	60 (61.9)	0.70

Cardiovascular risk factors				
Total cholesterol mmol/L	5.3 ± 0.1	5.1 ± 0.1	5.4 ± 0.1	0.23
Diabetes	16 (7.0)	5 (7.9)	2 (2.1)	0.17
Smoking	8 (3.5)	3 (4.8)	6 (6.3)	0.55
Framingham 10-yr absolute risk	9.5 ± 0.4	8.8 ± 0.7	9.1 ± 0.6	0.65

Psychological distress				
Depression	7.4 ± 0.5	7.9 ± 1.0	8.8 ± 0.8	0.37
Stress	14.5 ± 0.4	14.5 ± 0.8	15.6 ± 0.7	0.94

Readiness to change				
Exercise	3.6 ± 0.04	3.3 ± 0.1	3.5 ± 0.1	0.06
Diet	3.5 ± 0.04	3.5 ± 0.1	3.5 ± 0.1	0.84

**Table 3:** Correlations of psychological distress and readiness for change.

		*r *	*P* Value
Correlates of baseline exercise	Depression	−0.17	0.002
	Stress	−0.18	0.001

Correlates of baseline diet	Depression	−0.11	0.05
	Stress	−0.11	0.04

Correlates of Post-Intervention exercise*	Depression	−0.14	0.01
	Stress	−0.17	0.01

Correlates of Post-Intervention diet*	Depression	−0.08	0.15
	Stress	−0.05	0.37

*Partial correlations adjusted for baseline exercise, diet, depression, and stress.

**Table 4:** Readiness for change Post-Intervention.

	M ± SE or *N* (%)		
	0 e-mails (*n* = 227)	1–7 e-mails (*n* = 63)	≥8 e-mails (*n* = 97)
Exercise* after intervention	3.51 ± 0.04	3.62 ± 0.08	3.68 ± 0.07^†^
Diet* after intervention	3.60 ± 0.03	3.54 ± 0.06	3.73 ± 0.05^‡^

*Adjusted for baseline readiness to change exercise/diet, sex, age, antidepressant-anxiolytic medications, baseline depression, and stress.

Bonferroni post hoc comparison to 0 E-mail group: ^†^ *P* = 0.04, ^‡^ *P* = 0.02.
